# The New Era of Intraperitoneal Carboplatin in Ovarian Cancer: From Biological Rationale to Clinical Implementation

**DOI:** 10.3390/cancers18050764

**Published:** 2026-02-27

**Authors:** Shoji Nagao, Atsushi Fujikawa, Yui Tanaka, Momoko Tanioka, Ryoko Imatani, Yoshinori Tani, Hanako Sugihara, Kazuhiro Okamoto, Hirofumi Matsuoka, Naoyuki Ida, Junko Haraga, Chikako Ogawa, Hisashi Masuyama

**Affiliations:** Department of Obstetrics and Gynecology, Faculty of Medicine, Dentistry and Pharmaceutical Sciences, Okayama University, 2-5-1 Shikata-cho, Kita-ku, Okayama 700-8558, Japankazuhiro.errore@gmail.com (K.O.); masuyama@cc.okayama-u.ac.jp (H.M.)

**Keywords:** ovarian cancer, intraperitoneal chemotherapy, carboplatin, dose-dense TC therapy, homologous recombination deficiency, PARP inhibitor, neoadjuvant chemotherapy

## Abstract

Epithelial ovarian cancer is predominantly characterized by peritoneal dissemination, providing a strong biological rationale for intraperitoneal (IP) chemotherapy. Although IP cisplatin-based regimens have demonstrated substantial survival benefits in pivotal randomized trials, toxicity and catheter-related complications limit their widespread adoption. IP carboplatin has emerged as a pragmatic alternative with improved tolerability while preserving its pharmacokinetic advantages. This review summarizes the biological and pharmacological rationale for IP carboplatin and critically examines the clinical evidence, with a particular emphasis on the Intraperitoneal Carboplatin for Ovarian Cancer (iPocc) trial and its divergence from Gynecologic Oncology Group (GOG)-252. We further discuss the potential applicability of IP carboplatin beyond the traditional setting of minimal residual disease, including patients undergoing neoadjuvant chemotherapy and interval debulking surgery, as well as its possible use in the contemporary era of maintenance therapy. Collectively, the accumulated evidence supports renewed consideration of IP carboplatin as a versatile component in modern ovarian cancer management.

## Simple Summary

This review explores the expanding clinical role of intraperitoneal (IP) carboplatin in ovarian cancer, transcending the traditional “optimal debulking only” paradigm. Based on the iPocc trial, IP carboplatin shows promise across all residual tumor strata, including suboptimal cytoreduction and the neoadjuvant chemotherapy followed by interval debulking surgery setting. Mechanistically, “bidirectional” exposure—attaining high local concentrations while maintaining systemic levels comparable to intravenous delivery—supports efficacy even in the presence of larger residual disease. Additionally, we discuss the integration of IP therapy into the current PARP inhibitor era. Intensified IP platinum exposure may deepen the initial clinical response, particularly in tumors with homologous recombination deficiency, thereby optimizing the therapeutic landscape for subsequent maintenance. While further data on catheter complications and PARP inhibitor interactions are warranted, IP carboplatin may represent a viable intensification strategy for selected patients in contemporary ovarian cancer management.

## 1. Introduction

Epithelial ovarian cancer is predominantly a peritoneal disease, characterized by diffuse intraperitoneal (IP) dissemination, biological characteristic that has long supported the rationale for IP chemotherapy. The peritoneal–plasma barrier enables markedly higher local drug exposure within the peritoneal cavity while simultaneously limiting systemic absorption. Pharmacokinetic studies have demonstrated that the IP administration of platinum agents achieves peritoneal concentrations approximately 10–20 times higher than those attained via intravenous (IV) delivery, thereby ensuring sustained tumor exposure [1].

This pharmacokinetic advantage has translated into meaningful clinical benefits, as evidenced by a series of pivotal Gynecologic Oncology Group (GOG) trials. In particular, GOG-114 and GOG-172 demonstrated a considerable overall survival (OS) advantage with IP cisplatin-based chemotherapy in patients with optimally debulked advanced ovarian cancer [2,3]. Despite substantial treatment-related toxicity and lower completion rates, GOG-172 reported a notable median OS improvement. Importantly, long-term follow-up analyses have confirmed that this survival benefit persists beyond 10 years, particularly in patients who completed the planned IP treatment cycles [4].

Nevertheless, the clinical implementation of IP chemotherapy is hindered by toxicity, catheter-related complications, and limited tolerability, which are largely attributable to the use of IP cisplatin [5,6]. These challenges have prompted the exploration of carboplatin as an alternative IP platinum agent. Carboplatin offers a more favorable toxicity profile while maintaining antitumor efficacy, and its pharmacokinetic properties, including high IP drug concentrations, support its suitability for IP administration. Consequently, IP carboplatin has emerged as a pragmatic evolution of regional chemotherapy, aiming to preserve the pharmacokinetic and survival advantages of IP delivery while improving feasibility and treatment adherence. In this review, we summarize the historical development and biological rationale of IP chemotherapy and critically examines the role of IP carboplatin in the modern management of ovarian cancer.

## 2. Review Methodology

This manuscript is a narrative review synthesizing the biological rationale, pharmacokinetic evidence, and clinical trial data related to IP carboplatin therapy in ovarian cancer. A structured literature search was conducted using PubMed and major gynecologic oncology clinical trial databases, including GOG and NRG Oncology trial reports. Clinical trial data were extracted from published reports of GOG and NRG Oncology randomized trials rather than direct database analysis. Publications addressing pharmacokinetics of platinum agents, intraperitoneal chemotherapy, randomized clinical trials, and prospective studies evaluating IP carboplatin were prioritized. Particular emphasis was placed on pivotal randomized trials of IP chemotherapy (including GOG-114, GOG-172, GOG-252, and the iPocc trial), pharmacokinetic analyses of platinum delivery, and clinical studies examining the feasibility of IP carboplatin in contemporary treatment settings. Reference lists of relevant articles were also reviewed to identify additional key publications. Because this article is a narrative review of previously published studies, no original data collection or statistical analysis was performed, and all interpretations are based on publicly available evidence, with careful consideration of study design, patient populations, and reported clinical endpoints.

## 3. Historical Evolution of Intraperitoneal Chemotherapy in Ovarian Cancer

The biological rationale for IP chemotherapy is fundamentally based on the clinicopathological observation that epithelial ovarian cancer remains predominantly confined to the peritoneal cavity throughout most of its natural history. Pharmacokinetic modeling initiated in the late 1970s demonstrated that direct IP administration of cytotoxic agents could achieve a significant “pharmacological advantage,” defined by the ratio of the area under the concentration–time curve (AUC) in the peritoneal fluid versus the plasma [7].

### 3.1. The First Breakthrough: The GOG-104 Trial

The clinical efficacy of IP therapy was first established on a large scale by the landmark GOG-104 trial [8]. This Phase III study enrolled patients with Stage III EOC who had undergone optimal debulking (residual disease ≤ 2 cm) and compared IV cyclophosphamide and IV cisplatin (100 mg/m^2^) with IV cyclophosphamide and IP cisplatin (100 mg/m^2^). The IP arm demonstrated a statistically significant improvement in median OS compared to the IV arm (49 vs. 41 months; *p* = 0.02). This corresponded to a 24% reduction in the risk of death with a hazard ratio (HR) of 0.76, marking a critical paradigm shift in the regional management of ovarian cancer.

### 3.2. Solidifying the Evidence: GOG-114 and GOG-172

Following the success of GOG-104, subsequent trials were conducted under stricter surgical criteria (residual disease ≤ 1 cm) to further validate the IP approach. GOG-114 trial: This evaluated a regimen of 2 cycles of IV carboplatin (AUC = 9) induction followed by IV paclitaxel and IP cisplatin (100 mg/m^2^) [2]. It reported significant improvements in both progression-free survival (PFS) (28 vs. 22 months; *p* = 0.01) and OS (63 vs. 52 months; *p* = 0.05). However, the trial was critiqued for its complex induction strategy, which made it difficult to isolate the specific benefit of the IP route from the effects of overall dose intensification. GOG-172 Trial: Providing a more definitive comparison of administration routes, GOG-172 evaluated standard IV paclitaxel/cisplatin against a regimen of IV paclitaxel, IP cisplatin (100 mg/m^2^), and IP paclitaxel (60 mg/m^2^ on day 8) [3]. This trial demonstrated an unprecedented median OS advantage of 15.9 months (65.6 vs. 49.7 months; *p* = 0.03), with a HR for death of 0.73.

### 3.3. The Challenge of Toxicity and the “IP Paradox”

Despite these survival gains, IP therapy was associated with a significant toxicity profile that hindered its widespread adoption. In GOG-172, Grade 3–4 hematologic, gastrointestinal, and neurological toxicities were significantly more frequent in the IP arm [3]. Crucially, only 42% of patients in the IP arm were able to complete all six planned cycles [5]. The primary reasons for premature discontinuation were catheter-related complications, including infection, blockage, or leakage. This led to the emergence of the “IP Paradox”—the observation of a profound survival benefit despite low treatment compliance—suggesting that even a limited number of IP cycles can exert a powerful therapeutic effect on minimal residual disease.

### 3.4. Long-Term Outcomes and Dose–Response Relationship

Long-term follow-up of the GOG-114 and GOG-172 trials, with a median follow-up of 10.7 years, has confirmed the durability of the IP survival benefit. The median OS remained significantly longer in the IP group (61.8 vs. 51.4 months; HR 0.77) [4]. A clear dose–response relationship was observed, where each additional IP cycle completed was associated with a 12% reduction in the risk of death. Furthermore, the 5-year survival rate reached 59% for patients who completed 5–6 cycles, compared to only 18% for those who completed 1–2 cycles.

## 4. Pharmacokinetic Characteristics of Platinum Agents: IP Therapy as a Systemic Delivery Route (Table 1) [9]

The pharmacokinetic behavior of IP-administered anticancer agents depends largely on their molecular weight and hydrophilicity [10]. Platinum compounds, such as cisplatin and carboplatin, are low-molecular-weight water-soluble agents that are rapidly absorbed from the peritoneal cavity into the systemic circulation through capillary and lymphatic pathways [11,12]. Thus, although IP administration achieves markedly high regional drug concentrations, the systemic platinum exposure closely mirrors that achieved by IV delivery.

**Table 1:** Pharmacokinetic parameters.

Drug	Cisplatin	Carboplatin	Paclitaxel
Molecular mass	300.5	371.5	853.92
Water solubility	+	+	−
T_1/2_ peritoneum (h)	0.88	0.92	73.4
Peak ratio peritoneum/serum	20	16	
AUC ratio peritoneum/serum	12	18	996

T, time; AUC, area under the concentration—time curve.

Pharmacokinetic analyses have demonstrated that, following IP carboplatin administration, the 24 h area under the concentration–time curve (AUC) for serum platinum is nearly identical to that achieved with IV administration [13]. In contrast, peritoneal AUC remains approximately 15–20-fold higher with the IP route. This dual pharmacokinetic profile indicates that IP platinum therapy provides intensified regional exposure while maintaining systemic drug efficacy comparable to that of conventional IV administration.

Conversely, paclitaxel exhibits distinct pharmacokinetic properties. As a high-molecular-weight hydrophobic compound, paclitaxel is retained within the peritoneal cavity for prolonged periods, resulting in an exceptionally high peritoneal-to-plasma AUC ratio, often exceeding 1000 [14]. Although this property supports sustained local exposure, systemic absorption is limited and delayed. Furthermore, prolonged local retention of paclitaxel has been associated with increased peritoneal irritation and catheter-related complications, as documented in landmark trials such as GOG-172 [3].

Notably, the depth of direct drug penetration from the peritoneal surface into the tumor tissue is limited to a few millimeters (typically 1–3 mm) [15,16]. Therefore, the clinical efficacy of IP chemotherapy cannot be solely attributed to its direct diffusion into the peritoneal implants. For platinum-based agents, rapid systemic absorption facilitates secondary tumor exposure via the underlying vasculature (tumor-feeding blood vessels). This enables IP administration to function as a systemic treatment while simultaneously delivering high local concentrations to the serosal surface. This mechanistic synergy explains why IP platinum therapy remains effective even in patients with larger residual tumors, a finding supported by both historical GOG trials and more recent studies on IP carboplatin.

Overall, for rapidly absorbed agents such as platinum compounds, the IP route should be redefined not merely as a local treatment but also as a hybrid systemic delivery strategy.

## 5. IP Carboplatin for Ovarian Cancer with Large Residual Tumor Burden

Clinical evidence supports this conceptual framework. Long-term follow-up analyses of the GOG-114 and GOG-172 trials demonstrated that the survival benefit of IP therapy was not restricted to patients with minimal residual disease [4]. Importantly, improved outcomes were observed among patients who completed a greater number of IP cycles, suggesting that treatment completion and cumulative systemic platinum exposure, rather than residual tumor size alone, may be key determinants of benefit. Although these findings should be interpreted with caution owing to the potential selection bias, they suggest the possibility that IP platinum therapy may retain activity beyond microscopic disease, although the evidence remains indirect. Moreover, several retrospective and phase II studies evaluating IP carboplatin in combination with IV paclitaxel have demonstrated clinical activity in patients with suboptimal residual disease following primary cytoreductive surgery [17,18]. Collectively, these data support the feasibility of IP carboplatin in clinical settings traditionally considered less suitable for IP therapy.

**Figure 1:**
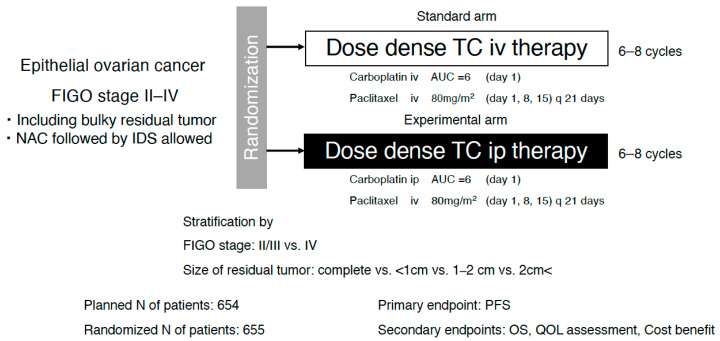
Schema of iPocc trial. AUC, area under the concentration–time curve; NAC, neoadjuvant chemotherapy; IDS, interval debulking surgery; PFS, progression-free survival; OS, overall survival; QOL, quality of life; IP, intraperitoneal; IV, intravenous.

More recently, the Intraperitoneal Carboplatin for Ovarian Cancer (iPocc) trial provided prospective randomized evidence that IP administration of carboplatin was superior to IV administration in patients with epithelial ovarian cancer, including those with larger residual tumors (Figure 1) [19]. Although statistically significant, the absolute improvement in median PFS was modest. Notably, this benefit was achieved with an acceptable safety profile, with port-related complications identified as the main additional toxicities. Therefore, the iPocc trial expanded the clinical applicability of IP chemotherapy by demonstrating that IP carboplatin can retain therapeutic efficacy across a broader spectrum of tumors than previously assumed.

Collectively, these pharmacokinetic and clinical data support IP carboplatin as a rational treatment option for selected patients with ovarian cancer, including those with relatively large residual tumor volumes. Rather than being strictly limited to patients with minimal residual disease, IP carboplatin may offer intensified peritoneal drug exposure without compromising systemic efficacy, provided that treatment delivery and completion can be adequately maintained. However, patients who complete a greater number of IP cycles likely represent a biologically and clinically favorable subset, introducing an inherent selection bias that limits causal interpretation.

## 6. Why Did GOG-252 Fail to Confirm Benefit While iPocc Was Positive? Differences in Populations and Trial Context (Table 2)

**Figure 2:**
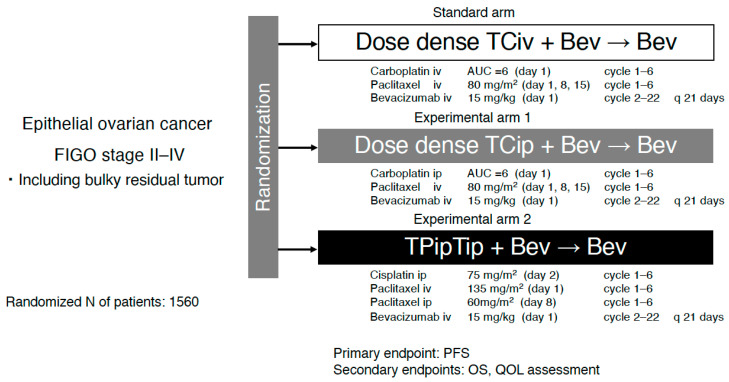
Schema of GOG-252 trial. AUC, area under the concentration–time curve; PFS, progression-free survival; OS, overall survival; QOL, quality of life; IP, intraperitoneal; IV, intravenous.

Direct comparisons between GOG-252 and iPocc should be interpreted cautiously because of differences in trial era, geographic population, and use of maintenance therapy. However, although both GOG-252 and iPocc evaluated IP carboplatin delivery within a modern paclitaxel–carboplatin backbone, their trial designs and clinical contexts were fundamentally distinct, likely contributing to their divergent outcomes (Figure 2) [19,20].

**Table 2:** Summary of results from the iPocc and GOG-252 trial.

		iPocc		GOG252		
		ddTCiv	ddTCip	ddTCiv + Bev	ddTCip + Bev	TPipTip + Bev
N		328	327	521	518	521
mPFS (months)		20.7	23.5	24.9	27.4	26.2
HR (95%CI)		Ref	0.83 (0.69–0.99)	Ref	0.925 (0.802–1.07)	0.977 (0.847–1.13)
mOS (months)		64.9	67	75.5	78.9	72.9
HR (95%CI)		Ref	0.95 (0.77–1.17)	Ref	0.949 (0.799–1.128)	1.05 (0.884–1.24)
Response (%)	CR	11.3	15.2	NR	NR	NR
	CR + PR	72.6	70.2	NR	NR	NR
FIGO stage (%)	II	14.0	12.8	10.7	10.8	9.8
	III	68.0	68.8	84.6	83.4	82.9
	IV	18.0	18.3	4.6	5.8	7.3
Residual disease (%)	Microscopic only	25.0	24.8	57.0	57.3	58.7
	0 < diameter ≤ 1 cm	14.3	15.3	34.9	36.5	34.9
	Dimameter > 1 cm	60.7	59.9	8.1	6.2	6.3

mPFS, median progression-free survival; mOS, median overall survival; NR, not recorded.

First, the use of bevacizumab differed substantially between the two trials. In GOG-252, bevacizumab was administered concomitantly and as a maintenance therapy across all treatment arms [20]. In this context, neither IP regimen (dose-dense TCip [ddTCip] or tri-weekly TCip) improved progression-free survival (PFS) or OS compared with the IV control arm (median PFS: 24.9 vs. 27.4 vs. 26.2 months; hazard ratio [HR] 0.925 and 0.977, respectively). In contrast, iPocc was designed to isolate the specific effect of the IP route by excluding bevacizumab and ensuring that all variables, except the administration route, remained identical (dose-dense TCiv [ddTCiv] vs. ddTCip). Although it remains hypothetical and unproven, this approach effectively minimizes confounding factors that may mask regional advantages of IP delivery.

Furthermore, there were notable differences in patient populations and surgical pathways. The iPocc trial enrolled patients with FIGO stage II–IV epithelial ovarian cancer and specifically addressed whether IP carboplatin could improve outcomes even in patients with suboptimal cytoreduction after primary debulking surgery (PDS) [19]. Patients undergoing interval debulking surgery (IDS) after neoadjuvant chemotherapy (NAC) were also included. This broader inclusion eligibility aligns with the evolving paradigm that, for rapidly absorbed platinum agents, IP administration functions as a hybrid strategy of regional intensification and systemic therapy and may benefit a wider range of patients than previously assumed.

Third, potential interaction between antiangiogenic therapy and IP chemotherapy warrants further investigation. Subgroup analyses of the GOG-252 trial suggested that bevacizumab may attenuate the relative benefit of IP carboplatin [20]. While the precise mechanism remains unclear, a plausible biological explanation is that bevacizumab may optimize peritoneal control by altering vascular permeability and drug distribution, thereby reducing the incremental advantage of IP carboplatin over IV counterpart and making a positive treatment signal more difficult to detect.

Finally, survival data from iPocc support the long-term efficacy of this approach [19]. In iPocc, IP carboplatin significantly improved PFS compared with IV administration (median PFS 23.5 vs. 20.7 months; HR 0.83; *p* = 0.041). Notably, the PFS curves exhibited sustained separation during long-term follow-up, suggesting that a subset of patients derived a durable benefit from IP delivery. Collectively, these discrepancies in design and context indicate that the “negative” results of GOG-252 do not refute the efficacy of IP carboplatin; rather, they may reflect a trial architecture in which universal bevacizumab use may have inadvertently diluted the route-specific therapeutic effect [20]. These findings should be interpreted as supportive but not definitive evidence for broader clinical adoption.

## 7. Unresolved Questions, Implementation Challenges, and Real-World Generalizability

Despite the compelling pharmacological rationale and supportive randomized data, several unresolved issues must be addressed before IP carboplatin can be broadly adopted in routine clinical practice.

### 7.1. Selection Bias and Treatment Completion

A major interpretative challenge in IP chemotherapy trials is the inherent selection bias associated with treatment completion. Long-term analyses of GOG-114 and GOG-172 demonstrated a clear association between the number of completed IP cycles and survival benefit [4]. However, treatment completion represents a post-randomization variable and may reflect favorable baseline characteristics, including superior performance status, lower tumor burden, or more indolent tumor biology. Consequently, although the iPocc trial demonstrated benefit across residual disease strata [19], it remains difficult to fully disentangle the true therapeutic contribution of the IP route from patient-related prognostic factors.

### 7.2. Lack of Biomarker Stratification

Most IP chemotherapy trials, including iPocc, were conducted before the routine incorporation of molecular profiling. No prospective study has yet reported outcomes stratified by BRCA1/2 mutation status or broader homologous recombination deficiency (HRD). As a result, the interaction between intensified regional platinum exposure and molecular vulnerability remains undefined. In the current era of biomarker-driven oncology, this represents a critical evidence gap.

### 7.3. Interaction with Bevacizumab and PARP Inhibitors

The compatibility of IP carboplatin with modern maintenance strategies remains uncertain. In GOG-252, universal bevacizumab administration may have attenuated route-specific benefits [20]. Whether anti-angiogenic therapy alters peritoneal drug distribution or vascular delivery dynamics remains speculative. Similarly, although intensified platinum exposure could theoretically enhance responsiveness to subsequent PARP inhibitor maintenance, this hypothesis has not been prospectively validated.

### 7.4. Generalizability and Surgical Expertise

The safety and reproducibility of IP carboplatin depend heavily on institutional infrastructure and surgical expertise. Port-related complications—including infection, obstruction, leakage, and catheter displacement—have historically been reported in 10–30% of patients [21]. Although carboplatin-based regimens demonstrate improved tolerability compared to cisplatin, technical complications remain a non-negligible barrier.

High-volume gynecologic oncology centers with established IP programs consistently report lower complication rates and higher completion rates than lower-volume institutions [22,23,24]. This raises concerns regarding the generalizability of trial results to broader practice settings. In addition, increasing use of neoadjuvant chemotherapy followed by interval debulking surgery may alter peritoneal anatomy through fibrosis or adhesion formation, potentially affecting catheter placement and drug distribution.

Collectively, these factors underscore that IP carboplatin should currently be considered a strategy best implemented within experienced centers capable of multidisciplinary coordination and structured port management protocols.

## 8. Expanding the Clinical Scope: NAC/IDS, Suboptimal Cytoreduction, and the PARP-Inhibitor Era

The design of the iPocc trial and subsequent subgroup observations support a broader conceptualization of IP carboplatin, moving beyond the traditional “optimal debulking only” paradigm [19]. Subgroup analyses suggested favorable outcomes with ddTCip across all residual tumor strata, indicating that the regimen remains a viable consideration even for patients planned for NAC followed by IDS. This expansion is clinically relevant, as real-world practice increasingly incorporates NAC followed by IDS strategies, particularly when complete upfront resection is not feasible.

### 8.1. Suboptimal Residual Disease or NAC Settings

The iPocc trial is unique in explicitly testing whether IP carboplatin improves outcomes in patients with suboptimal cytoreduction after PDS [19]. Mechanistically, this is supported by platinum pharmacokinetics, as rapid peritoneal absorption yields systemic exposure comparable to IV delivery while maintaining markedly higher IP concentrations. This “bidirectional” tumor exposure, occurring via both the peritoneal surface and the tumor vasculature, provides a biologically plausible basis for efficacy even in the presence of larger residual tumor implants. Patients with suboptimal residual disease may therefore be good candidates for IP carboplatin therapy, including chemotherapy administered as NAC.

### 8.2. NAC Followed by IDS Settings

Conceptually, NAC followed by IDS may markedly reduce the tumor burden and enhance the regional efficacy of IP therapy by minimizing drug diffusion distances [25,26]. Concurrently, IP platinum is rapidly absorbed into the systemic circulation, providing systemic exposure comparable to that achieved by IV administration. Consequently, if catheter/port placement is feasible and patient tolerability is maintained, IP carboplatin represents a reasonable intensification strategy following IDS. Nevertheless, it remains unclear whether surgical alterations or adhesions resulting from IDS increase the risk of catheter-related complications. Further ancillary analyses and prospective studies are needed to address these uncertainties. 

### 8.3. Molecular Rationale: Platinum Sensitivity, Homologous Recombination Deficiency, and Intraperitoneal Intensification

Platinum agents exert their antitumor effects by forming DNA intra-strand and inter-strand crosslinks [27]. These lesions physically obstruct replication fork progression, leading to fork collapse and the generation of lethal DNA double-strand breaks. In healthy cells, these double-strand breaks are accurately repaired via homologous recombination, a process dependent on functional proteins such as BRCA1 and BRCA2 [28]. Conversely, cells with HRD must rely on error-prone repair pathways, resulting in profound genomic instability and apoptosis. This defect establishes the molecular vulnerability that defines high platinum sensitivity in a subset of ovarian cancers.

The high responsiveness of HRD-positive tumors provides a compelling rationale for the regional dose intensification achieved by IP delivery. IP administration yields peritoneal platinum concentrations 10 to 20 times higher than intravenous delivery [10]. This intensified exposure may completely saturate the limited repair capacity of HRD-deficient cells, maximizing the cumulative DNA damage burden. Retrospective analyses confirmed that *BRCA1*-deficient patients derived the greatest benefit from IP therapy. In this cohort, the median OS reached 84 months with the IP regimen compared to 47 months with standard IV therapy [29]. The profound cytoreduction achieved by IP intensification may serve as an ideal foundation for subsequent maintenance with PARP inhibitors. Furthermore, the transition toward IP carboplatin, as investigated in the iPocc trial, aims to leverage these regional advantages while offering a superior toxicity profile compared to cisplatin-based protocols [19].

### 8.4. Positioning Alongside Poly(ADP-Ribose) Polymerase (PARP) Inhibitors

A pivotal unresolved issue is whether the clinical benefits of IP carboplatin are preserved when integrated with modern maintenance strategies, specifically PARP inhibitors. Although current evidence has yet to define the impact of this interaction, necessitating rigorous, randomized phase III trials, a compelling conceptual rationale for this combination exists. Tumors characterized by homologous recombination deficiency, including those with *BRCA1/2* mutations, exhibit marked sensitivity to both platinum agents and PARP inhibition [29,30]. Consequently, intensified IP platinum exposure may augment the depth of the initial clinical response, potentially optimizing the therapeutic landscape for subsequent PARP maintenance and extending the duration of disease control. This concept remains biologically plausible but clinically unproven.

### 8.5. Should We Use Dose-Dense TC Therapy or Bevacizumab Combination Maintenance Therapy (Table 3)?

Across three large randomized trials comparing conventional triweekly paclitaxel plus carboplatin (TC) with either ddTC or TC combined with bevacizumab followed by bevacizumab maintenance—namely JGOG3016, GOG-218, and ICON7—distinct and clinically relevant patterns of treatment effect have been observed [31,32,33]. In JGOG3016, ddTC was associated with a significant OS benefit that appeared largely independent of established clinical risk factors. In contrast, in GOG-218 and ICON7, the addition of bevacizumab followed by maintenance therapy yielded the greatest benefit in patients with high-risk disease, whereas its impact was attenuated or absent in patients with lower-risk features. These findings collectively suggest that intensified paclitaxel delivery through a dose-dense schedule may exert antitumor effects with relatively limited dependence on baseline clinical risk stratification, whereas the benefit of anti-angiogenic therapy using bevacizumab is more context-dependent. Within this framework, the integration of IP carboplatin into a ddTC backbone represents a biologically and clinically coherent strategy. Given that dose-dense paclitaxel appears to provide consistent benefit across a broad clinical spectrum, ddTCip may be applicable to a wide range of patients without substantial modification based on conventional clinical risk factors. This contrasts with bevacizumab-containing strategies, which may require more selective application based on disease burden and risk profile. Therefore, ddTCip may represent a broadly applicable intensification strategy, whereas bevacizumab-containing therapy may be more appropriate for selected high-risk patients.

**Table 3:** The impact of clinical risk factors on the additional OS benefit of dose-dense TC therapy and bevacizumab.

		JGOG3016	GOG218	ICON7
Control arm		triweekly TC	triweekly TC	triweekly TC
Experimental arm		dose dense TC	TC + Bev followed by Bev	TC + Bev followed by Bev
whole patients	HR	0.79	0.96	0.99
	95%CI	0.63–0.99	0.85–1.09	0.85–1.14
low-risk patients	HR	0.76	1.05	1.14
	95%CI	0.49–1.19	0.92–1.20	0.93–1.40
high-risk patients	HR	0.75	0.75	0.78
	95%CI	0.57–0.97	0.59–0.95	0.63–0.97

OS, overall survival; TC, paclitaxel + carboplatin; Bev, bevacizumab; HR, hazard ratio.

### 8.6. Practical Considerations

From a practical standpoint, successful implementation of IP carboplatin requires standardized port placement techniques, close monitoring of port-related complications, and patient education. Catheter-related complications have been reported in approximately 10–30% of patients in prior IP chemotherapy trials [21]. When these elements are appropriately addressed, IP carboplatin demonstrates high treatment adherence and a safety profile comparable to that IV chemotherapy, with the added advantage of enhanced peritoneal drug exposure.

## 9. Future Directions

Given the well-established biological interplay between platinum sensitivity and HRD, the clinical efficacy of first-line chemotherapy combining ddTCip followed by PARP inhibitor maintenance therapy warrants urgent prospective investigation. Such trials should incorporate comprehensive biomarker analyses, including BRCA1/2 mutation status and broader HRD profiling, to determine whether intensified platinum exposure during induction therapy can enhance the depth of initial response and, in turn, improve the magnitude and durability of benefit from subsequent PARP inhibitor-based maintenance therapy. Although this hypothesis remains unproven, it is biologically plausible that enhanced intraperitoneal and systemic platinum exposure achieved through IP carboplatin could optimize the therapeutic context for PARP inhibition, particularly in HRD-positive tumors. Carefully designed clinical trials integrating molecular stratification will therefore be essential to define the precise role of IP carboplatin within contemporary, biomarker-driven treatment algorithms for ovarian cancer.

## 10. Conclusions

IP carboplatin represents a biologically rational evolution of platinum-based chemotherapy for ovarian cancer, integrating intensified regional drug exposure with preserved systemic delivery in appropriately selected patients. Evidence from randomized trials, particularly the iPocc study, demonstrates that IP carboplatin within a dose-dense paclitaxel backbone can achieve a statistically significant improvement in PFS, although the absolute magnitude of benefit is modest and OS data remain immature.

Importantly, current evidence does not support universal adoption of IP carboplatin as a standard of care. Rather, its clinical value appears context-dependent. The potential benefit may be most relevant in patients treated within a dose-dense paclitaxel framework, particularly in settings where bevacizumab is not routinely incorporated. Moreover, the reproducibility of outcomes outside specialized, high-volume centers remains uncertain.

In the modern therapeutic landscape, critical unanswered questions include molecular stratification by HRD status, integration with PARP inhibitor maintenance therapy, and validation across diverse geographic populations. Until these issues are resolved through prospective biomarker-driven trials, IP carboplatin should be regarded as a rational intensification strategy for carefully selected patients treated at experienced institutions.

Future studies integrating molecular profiling and contemporary maintenance paradigms will be essential to define the precise positioning of IP carboplatin within evolving ovarian cancer treatment algorithms.

## Data Availability

No new data were created or analyzed in this study. Data sharing is not applicable to this study.

## Abbreviations

The following abbreviations are used in this manuscript:

AUC	Area under the concentration–time curve
GOG	Gynecologic Oncology Group
IDS	Interval debulking surgery
IP	Intraperitoneal
IV	Intravenous
NAC	Neoadjuvant chemotherapy
OS	Overall survival
PARP	Poly(ADP-ribose) polymerase
PDS	Primary debulking surgery
PFS	Progression-free survival
QOL	Quality of life
